# Supplementary material for: Targeting immune checkpoints potentiates immunoediting and changes the dynamics of tumor evolution
Source: Nat Commun. 2018 Jan 2;9:32. doi: 10.1038/s41467-017-02424-0 (PMC5750210; doi:10.1038/s41467-017-02424-0)
Supplement: Supplementary file 1 — Supplementary Information [file 41467_2017_2424_MOESM1_ESM.pdf]

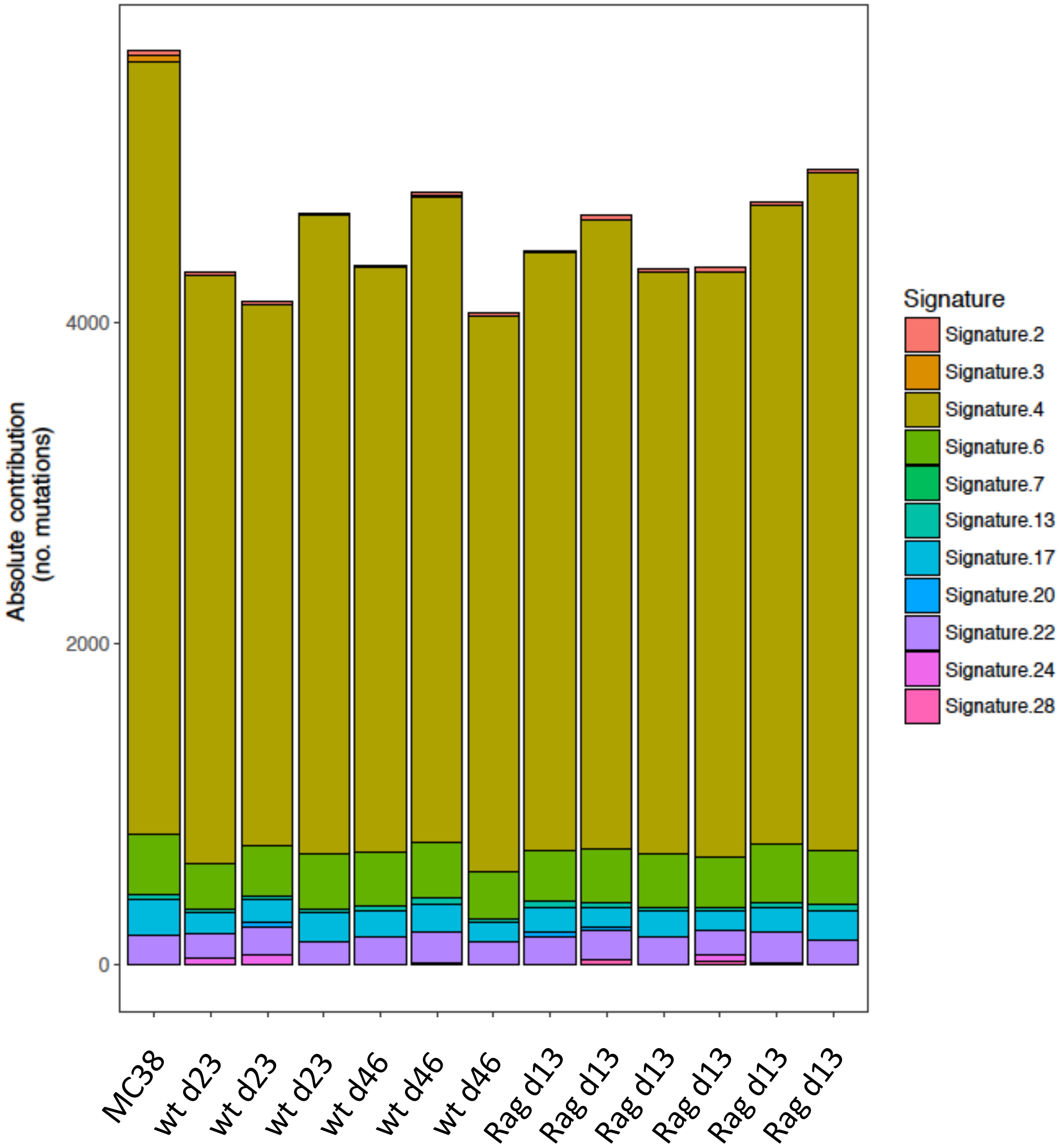

Supplementary Figure 1. The contributions of published mutational signatures from Alexandrov et al. Nature 500, 415-421 (2013) to individual MC38, wild type and RAG1$^{-/-}$ samples. The most prevalent signatures across all samples are Signature 3 (BRCA1/2 mutations), Signature 4 (Smoking) and Signature 6 (DNA MMR deficiency).

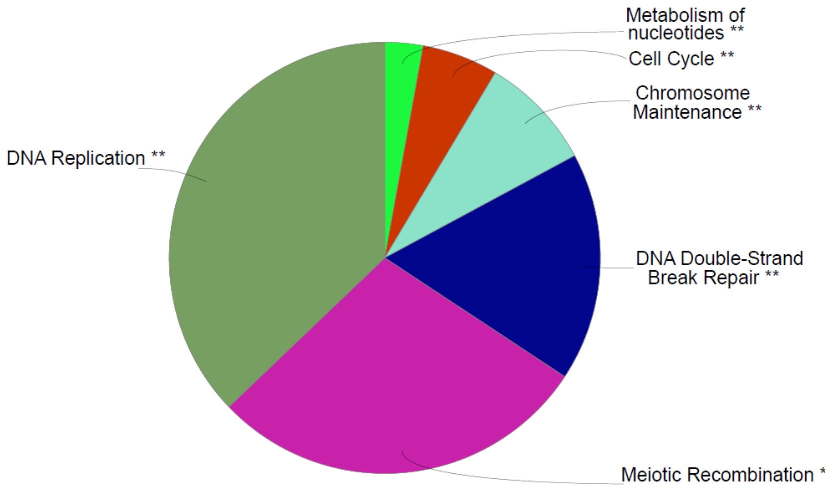

Supplementary Figure 2. Enriched pathways of the significantly upregulated genes in the MC38 cell line vs normal skin samples. The pie chart is created using ClueGO and shows the enriched groups (the name of the group is given by the most significant term). The sizes of the sections correlate with the number of terms included in a group.

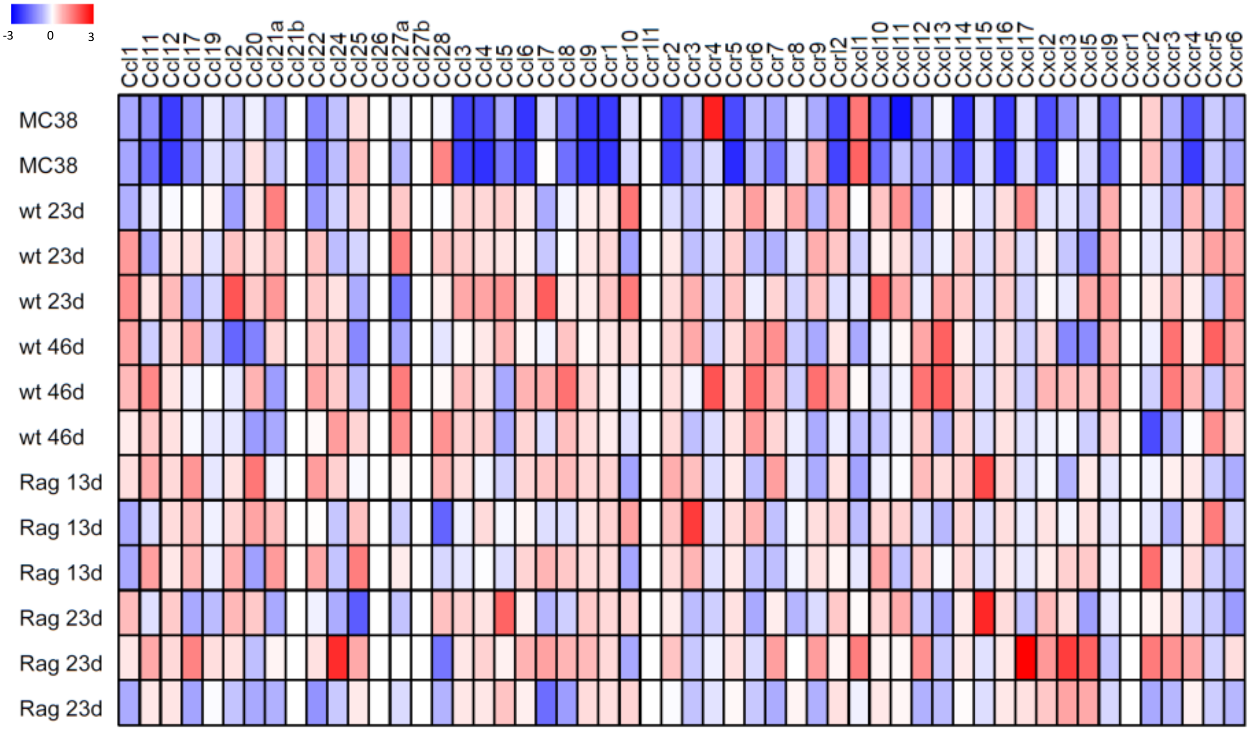

Supplementary Figure 3. z-score heatmaps of the log2 transformed normalized counts of chemokines and chemokine receptors in MC38, wild type and RAG1$^{-/-}$ samples.

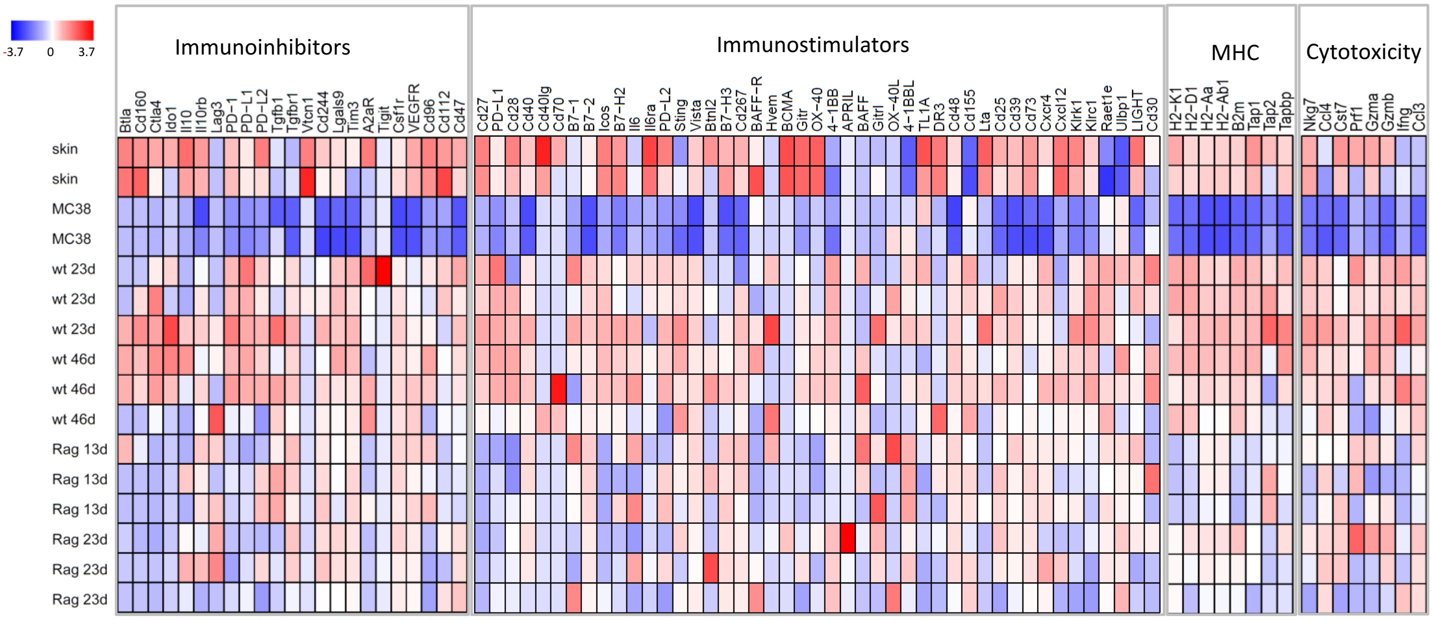

Supplementary Figure 4. z-score heatmaps of the log2 transformed normalized counts of selected genes in MC38, skin, wild type and RAG1$^{-/-}$ samples.

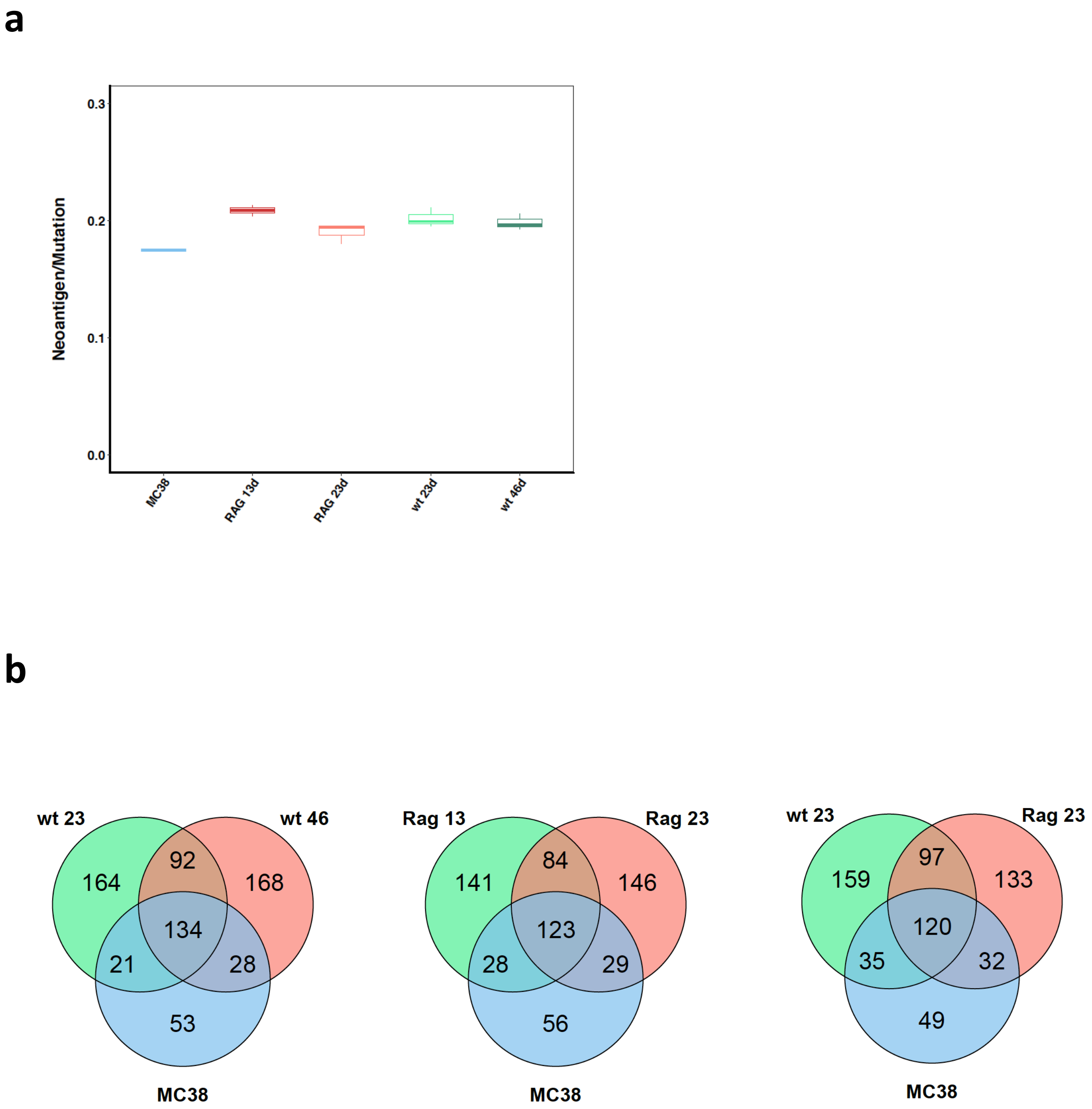

Supplementary Figure 5. a) Ratio between the number of expressed neoantigens and nonsynonymous mutations in MC38, Rag 13d, Rag 23d, wt 23d, wt 46d (n=3 replicates for all samples expect MC38 where n=1). Box plots show the median, the 25th and 75th percentiles. b) Shared LOH events between MC38, wild type and RAG1$^{-/-}$ samples. LOH events found in at least one sample from the same type are considered.

a

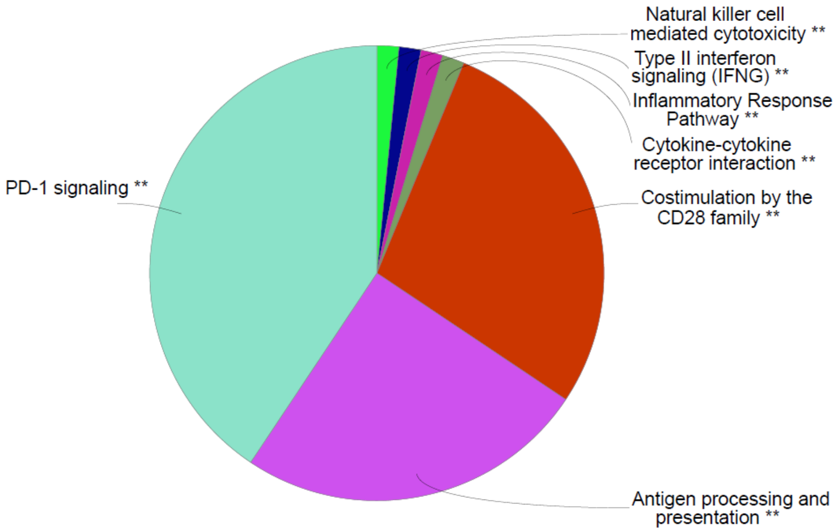

b

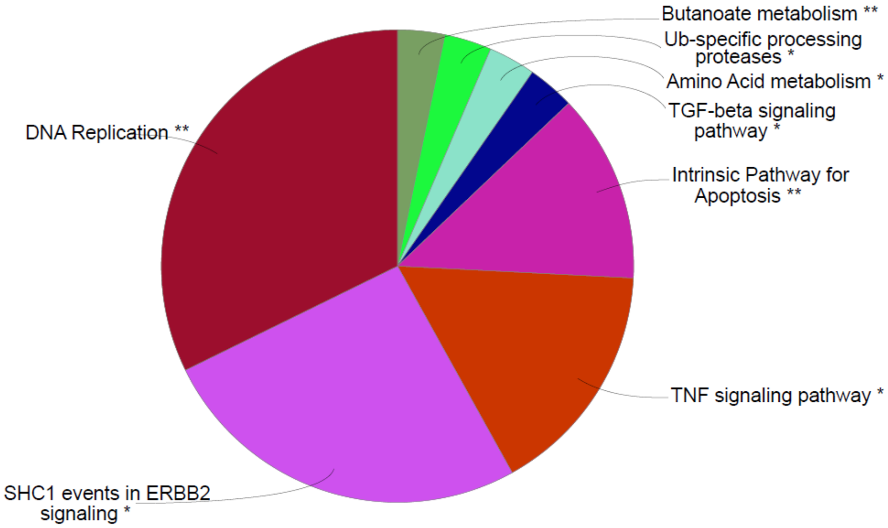

Supplementary Figure 6. Enriched pathways of the significantly a) upregulated and b) downregulated genes in wild type day 23 vs RAG1$^{-/-}$ day 23 tumors. The pie chart is created using ClueGO and shows the enriched groups (the name of the group is given by the most significant term). The sizes of the sections correlate with the number of terms included in a group.

242	clonal	subclonal	not shared
S1	75	43	124
S2	81	75	86
S3	81	85	76
S4	91	65	86
S5	77	50	115
s6	76	63	103

110	clonal	sub clonal	not shared
S1	22	17	71
S2	14	14	82
S3	31	30	49

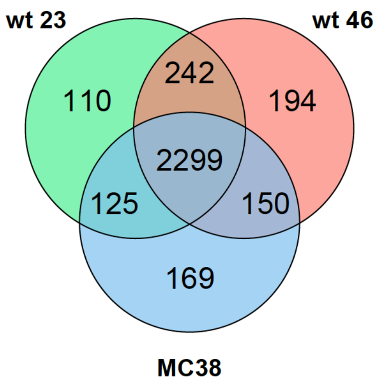

194	clonal	sub clonal	not shared
S1	37	33	124
S2	39	59	96
S3	14	37	143

125	clonal	sub clonal	not shared
S1	40	19	66
S2	22	16	87
S3	31	35	59
S4	54	64	7

150	clonal	sub clonal	not shared
S1	39	11	100
S2	60	45	45
S3	27	23	100
S4	52	93	5

Supplementary Figure 7. Number of clonal / subclonal shared nonsynonymous mutations between MC38 and wild type samples during progression. Each circle represents union of mutations across three replicates.

230	clonal	subclonal	not shared
S1	87	56	87
S2	81	48	101
S3	78	60	92
S4	91	42	97
S5	69	55	106
s6	88	64	78

95	clonal	sub clonal	not shared
S1	18	11	66
S2	42	13	40
S3	14	21	60

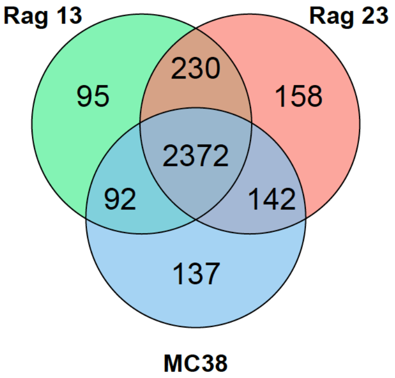

158	clonal	sub clonal	not shared
S1	29	10	119
S2	40	33	85
S3	49	27	82

92	clonal	sub clonal	not shared
S1	18	25	49
S2	23	17	52
S3	17	30	45
S4	28	61	3

142	clonal	sub clonal	not shared
S1	39	21	82
S2	33	46	63
S3	35	51	56
S4	53	89	0

Supplementary Figure 8. Number of clonal / subclonal shared nonsynonymous mutations between MC38 and RAG1$^{-/-}$ samples during progression. Each circle represents union of mutations across three replicates.

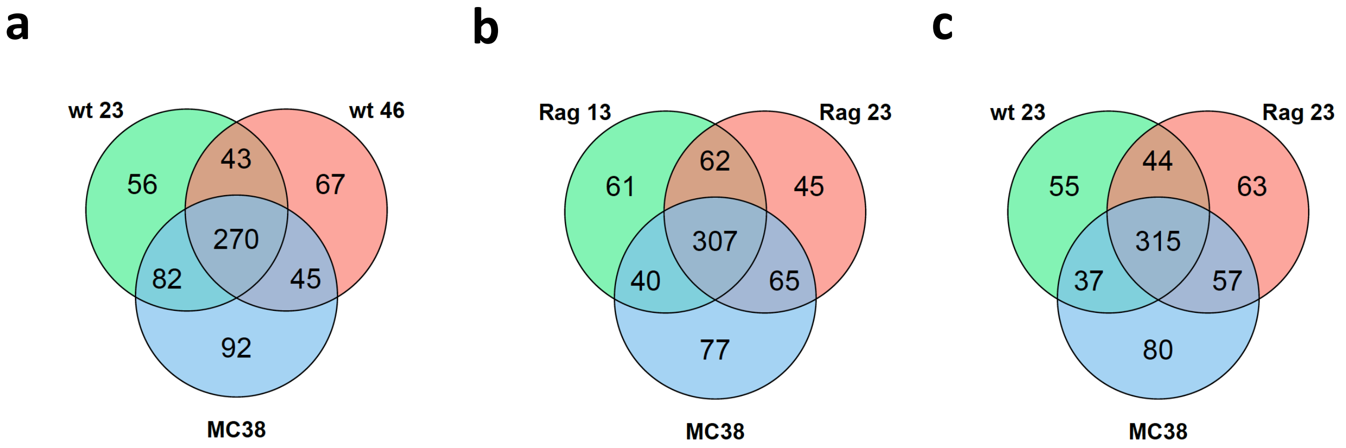

Supplementary Figure 9. Shared expressed neoantigens between MC38 and individual replicates of wild type and RAG1$^{-/-}$ samples.

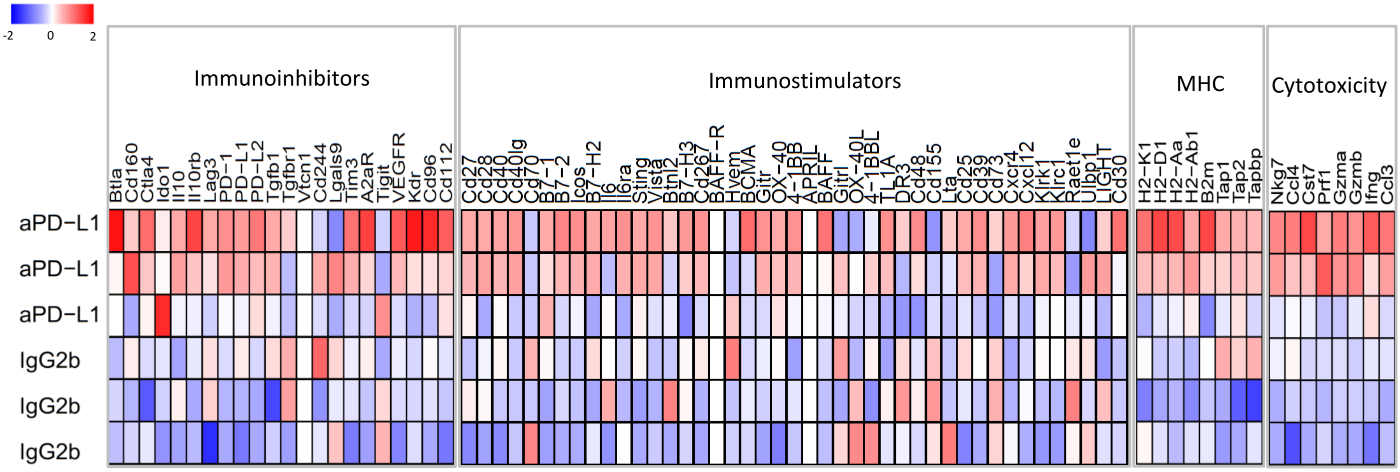

Supplementary Figure 10. z-score heatmaps of the log2 transformed normalized counts of selected genes in the anti-PD-L1 and the IgG2b control samples.

a

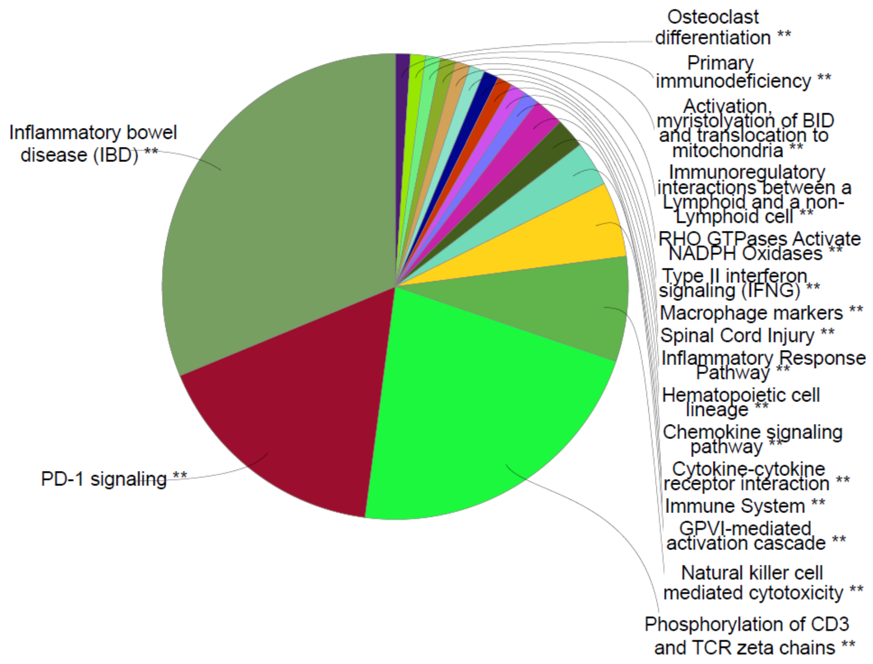

b

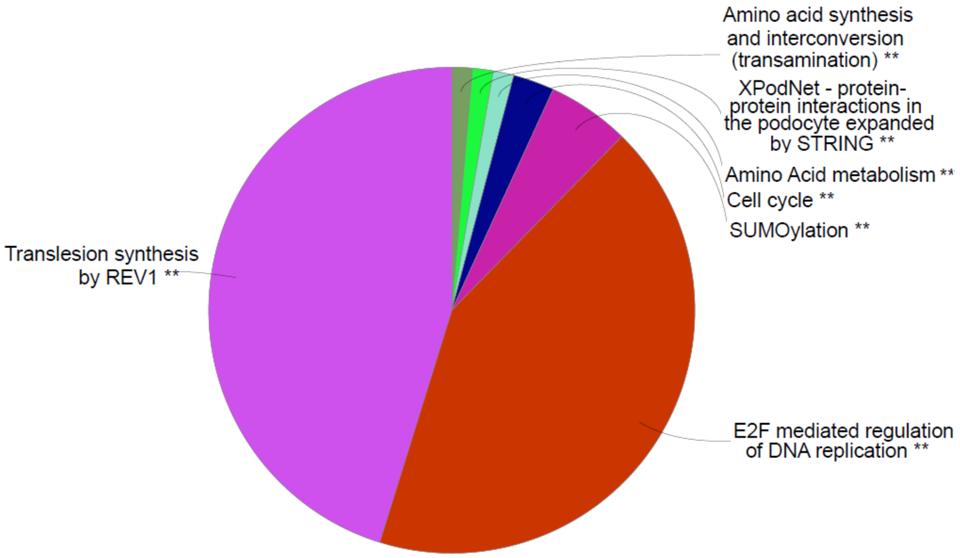

Supplementary Figure 11. Enriched pathways of the significantly a) upregulated and b) downregulated genes in the antiPD-L1 vs the IgG2b tumors. The pie chart is created using ClueGO and shows the enriched groups (the name of the group is given by the most significant term). The sizes of the sections correlate with the number of terms included in a group.

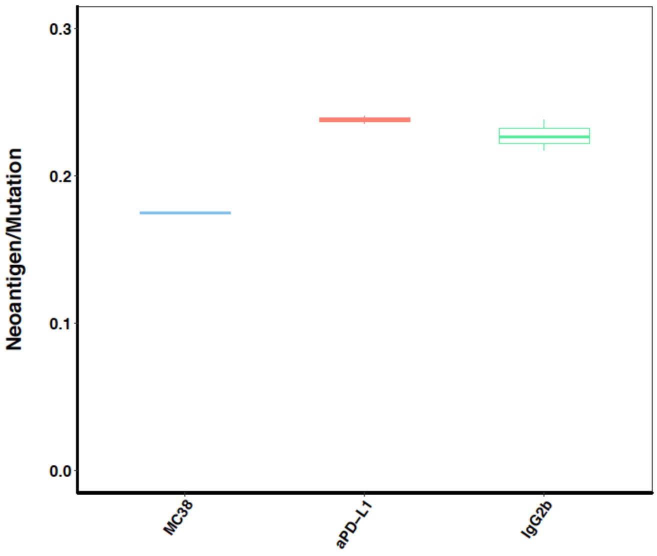

Supplementary Figure 12. Ratio between the number of expressed neoantigens and nonsynonymous mutations in MC38, anti-PD-L1 (n = 2) and IgG2b samples (n=3). Box plots show the median, the 25th and 75th percentiles.

115	clonal	subclonal	not shared
S1	64	13	38
S2	66	15	34
S3	75	7	33
S4	47	1	67
S5	90	11	14

22	clonal	sub clonal	not shared
S2	6	0	16
S3	13	5	4

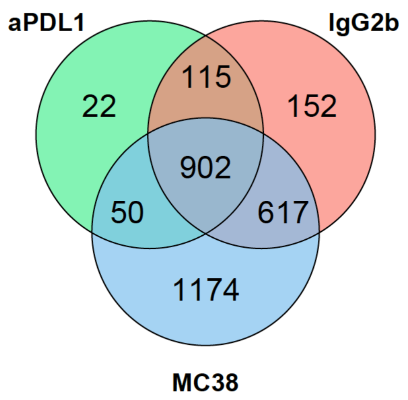

152	clonal	sub clonal	not shared
S1	68	6	78
S2	61	13	78
S3	57	19	76

50	clonal	sub clonal	not shared
S1	8	0	42
S2	43	1	6
S3	29	20	1

617	clonal	sub clonal	not shared
S1	313	14	290
S2	309	28	280
S3	322	36	259
S4	363	238	16

1174	clonal	subclonal	filtered or no CNA
S1	509	644	21

902	clonal	subclonal	not shared
S1	694	54	154
S2	705	56	141
S3	706	58	138
S4	462	13	427
S5	765	52	85
s6	713	161	28

Supplementary Figure 13. Number of clonal / subclonal shared nonsynonymous mutations between MC38, anti-PD-L1 and IgG2b samples during progression. Each circle represents union of mutations across three replicates.

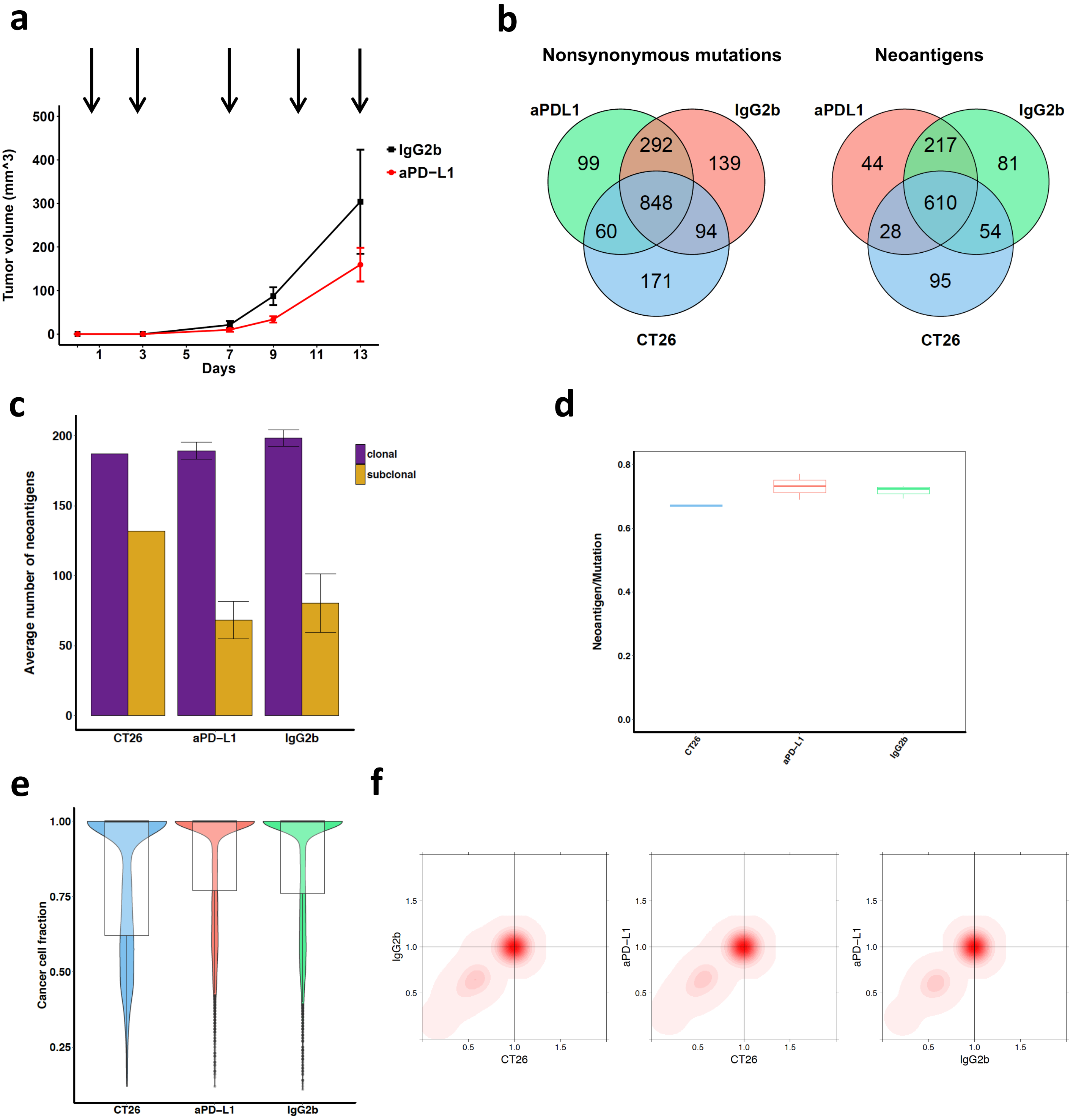

Supplementary Figure 14. Genomic and immunogenomic impact of targeting the PD-1/PD-L1 axis. a) Tumor growth curve in anti-PD-L1 treated mice (n=10) compared to tumor growth curve in mice (n=4) injected with IgG2b control. Presented is the mean ± SEM. b) Shared nonsynonymous mutations and neoantigens between CT26, and anti-PD-L1 treated and control samples. Mutations/neoantigens found in at least one sample from the same type are considered. c) Fractions of clonal and subclonal neoantigens in MC38 and all tumor samples (n=3).; error bars represent SEM. d). Ratio between the number of neoantigens and nonsynonymous mutations in CT26, anti-PD-L1 (n=3) and IgG2b (n=3) samples. Box plots show the median, the 25th and 75th percentiles. E) Violin plots showing tumor heterogeneity estimated from the cancer cell fractions (n=3 replicates for all samples expect for CT26 where n=1). f) Two-dimensional density plots showing the clustering of the cancer cell fractions of all mutations shared between two samples

292	clonal	subclonal	not shared
S1	104	117	71
S2	93	73	126
S3	97	58	137
S4	108	102	82
S5	110	66	116
s6	77	41	174

99	clonal	sub clonal	not shared
S1	39	14	46
S2	22	5	72
S3	25	4	70

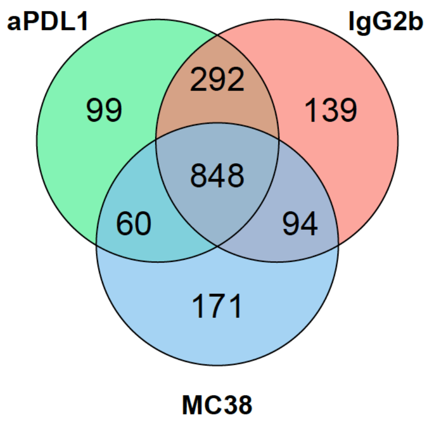

139	clonal	sub clonal	not shared
S1	50	43	46
S2	30	9	100
S3	25	8	106

60	clonal	sub clonal	not shared
S1	25	12	23
S2	14	4	42
S3	11	1	48
S4	33	26	1

94	clonal	sub clonal	not shared
S1	42	29	23
S2	17	11	66
S3	20	12	62
S4	32	59	3

171	clonal	subclonal	filtered or no CNA
S1	60	104	7

848	clonal	subclonal	not shared
S1	565	198	85
S2	525	123	200
S3	530	126	192
S4	575	185	88
S5	506	125	217
s6	498	113	237
s7	569	256	23

Supplementary Figure 15. Number of clonal / subclonal shared nonsynonymous mutations between CT26, anti-PD-L1 and IgG2b samples during progression. Each circle represents union of mutations across three replicates.

Supplementary Table 1. Number of mutations that changed their variant allele frequencies (VAF) and cancer cell fractions (CCF) between time points in the three wt and RAG1$^{-/-}$ samples out of the total 2299 (shared between wt23 and wt46) and 2372 (shared between RAG1$^{-/-}$13 and RAG1$^{-/-}$23) mutations.

wt23 vs wt46 diff in VAF > 0.2	wt23 vs wt46 diff in CCF > 0.3	RAG1$^{-/-}$13 vs RAG1$^{-/-}$23 diff in VAF > 0.2	RAG1$^{-/-}$ vs RAG1$^{-/-}$23 diff in CCF > 0.3
71	75	83	67
102	98	79	68
87	90	61	77
