# Supplementary material for: Targeting immune checkpoints potentiates immunoediting and changes the dynamics of tumor evolution
Source: Nat Commun. 2018 Jan 2;9:32. doi: 10.1038/s41467-017-02424-0 (PMC5750210; doi:10.1038/s41467-017-02424-0)
Supplement: Supplementary file 3 — Description of Additional Supplementary Files [file 41467_2017_2424_MOESM3_ESM.pdf]

Description of Additional Supplementary Files

File Name: Supplementary Data 1
Description: Driver genes in MC38 and mutational hotspots in human CRC.

File Name: Supplementary Data 2
Description: List of expressed predicted neoantigens present in the MC38 cell line and the RAG1-/- samples from day 23, and absent from the wild type samples from day 23.

File Name: Supplementary Data 3
Description: List of expressed predicted neoantigens present in the MC38 cell line and the IgG2b control samples, and absent from the anti-PD-L1 samples.

File Name: Supplementary Data 4
Description: List of expressed predicted neoantigens present in the CT26 cell line and the IgG2b control samples, and absent from the anti-PD-L1 samples.
